# Bisphenol-A and phthalate metabolism in children with neurodevelopmental disorders

**DOI:** 10.1371/journal.pone.0289841

**Published:** 2023-09-13

**Authors:** T. Peter Stein, Margaret D. Schluter, Robert A. Steer, Xue Ming

**Affiliations:** 1 Department of Surgery, Rowan University-School of Osteopathic Medicine, Stratford, NJ, United States of America; 2 Department of Psychiatry, Rowan University-School of Osteopathic Medicine, Stratford, NJ, United States of America; 3 Departments of Neurosciences and Neurology, Rutgers University–New Jersey Medical School, Newark, NJ, United States of America; King Faisal Specialist Hospital and Research Center, SAUDI ARABIA

## Abstract

**Background:**

The etiology of autism spectrum (ASD) and Attention Deficit/Hyperactivity (ADHD) disorders are multifactorial. Epidemiological studies have shown associations with environmental pollutants, such as plasticizers. This study focused on two of these compounds, the Bisphenol-A (BPA) and Diethylhexyl Phthalate (DEHP). The major pathway for BPA and DEHP excretion is via glucuronidation. Glucuronidation makes insoluble substances more water-soluble allowing for their subsequent elimination in urine.

**Hypothesis:**

Detoxification of these two plasticizers is compromised in children with ASD and ADHD. Consequently, their tissues are more exposed to these two plasticizers.

**Methods:**

We measured the efficiency of glucuronidation in three groups of children, ASD (n = 66), ADHD (n = 46) and healthy controls (CTR, n = 37). The children were recruited from the clinics of Rutgers-NJ Medical School. A urine specimen was collected from each child. Multiple mass spectrometric analyses including the complete metabolome were determined and used to derive values for the efficiency of glucuronidation for 12 varied glucuronidation pathways including those for BPA and MEHP.

**Results:**

(1) Both fold differences and metabolome analyses showed that the three groups of children were metabolically different from each other. (2) Of the 12 pathways examined, only the BPA and DEHP pathways discriminated between the three groups. (3) Glucuronidation efficiencies for BPA were reduced by 11% for ASD (p = 0.020) and 17% for ADHD (p<0.001) compared to controls. DEHP showed similar, but not significant trends.

**Conclusion:**

ASD and ADHD are clinically and metabolically different but share a reduction in the efficiency of detoxification for both BPA and DEHP with the reductions for BPA being statistically significant.

## Introduction

The etiology of autism spectrum disorders (ASD) is believed to be multifactorial. One prominent hypothesis involves toxicant exposure acting upon genetically susceptible individuals. ASD has strong associations with both environmental factors and genetic components [1–7]. It is now generally accepted that chemicals introduced into the environment by human activity can have adverse effects on human health [1, 8–12].

The present study focused on two of these compounds, the common plasticizers Bisphenol-A (BPA) and Diethylhexyl Phthalate (DEHP). BPA and phthalates plasticizers are moderate molecular weight, hydrophilic and relatively inert aromatic compounds. BPA is used in the manufacture of polycarbonate plastics, as an antioxidant in some plasticizers, in polyvinyl chloride (PVC) manufacture, and the Epoxy resins used to coat the inside of many food and beverage cans [11, 13, 14]. DEHP is used to control the rigidity of a plastic [7, 15]. The principal routes of exposure are believed to be dietary—through ingestion of food products via contaminated packaging although there is some evidence that inhalation and personal products are also important [12, 15, 16].

The evidence for an association of neurodevelopmental disorders with exposure to plasticizers is primarily from epidemiological studies based on maternal urine analyses [7]. Multiple mechanisms have been proposed; most involve the plasticizer acting as a weak endocrine disruptor [17–21]. Numerous gene mutations have also been associated with ASD [5, 6]. Details about how gene mutations lead to ASD in some children and not others are lacking.

We propose that the linkage is a genetically determined compromised ability to detoxify the plasticizers BPA and MEHP. The major pathway for BPA and DEHP metabolism and excretion is via glucuronidation. The glucuronidation pathway makes a large variety of substances more water-soluble allowing for their subsequent elimination from the body upon urination. Most BPA and DEHP excretion is as the glucuronide with a small amount as the sulfate [22–25].

The key step in the glucuronidation process is the transfer of glucuronic acid from uridine-5′-diphospho-α-D-glucuronic acid (UDPGA) to the target molecule which could be a hydroxyl, amine, carboxyl, sulfhydryl etc. by Uridine 5′-diphospho-glucuronosyltransferases (UGTs) [26–28]. The different UGT’s have numerous and overlapping substrates [29]. Thus, while the mechanism is common, execution is by a multitude of closely related enzymes leaving much scope for individual variability.

Previously, we reported that the efficiency of glucuronidation of DEHP and BPA metabolites was lower in children with ASD [30]. The objectives of the present study were:

1. To determine whether the association of compromised glucuronidation was specific to ASD or was more general applying to other childhood neurodevelopmental disorders by measuring the efficiency of glucuronidation for another neurological disorder. Attention Deficit/Hyperactivity disorder (ADHD) was selected as the other disorder because it has many outward similarities to ASD [31, 32]. ADHD is one of the most common neurodevelopmental disorders in children [33, 34]. Both BPA and phthalates have been implicated in the development of ADHD symptoms [12, 17, 35–37]. The prevalence of ADHD and ASD is higher in males than in females [33, 35, 38].
2. Glucuronidation encompasses many different sub-pathways. The second objective of the present study was to ascertain whether the association of ASD with decreased glucuronidation efficiency was unique to a specific plasticizer sub-pathway or common to other variants of the glucuronidation pathway. Therefore, the present study examined the relationships of ASD and ADHD with a varied group of twelve glucuronidation sub-pathways. Five were plasticizers, specifically, BPA and four phthalates, the primary metabolites of DEHP, mono-2-ethylhexyl phthalate (MEHP) and three of its secondary metabolites, mono-(2-ethyl-5-oxohexyl) phthalate (5-oxo MEHP), mono-(2-ethyl-5-hydroxyhexyl) phthalate (5-OH MEHP) and mono-(2-ethyl-5-carboxypentyl phthalate (5-CX MEPP). The other seven pathways were derived from metabolites present in the metabolome There was one steroid, cortisol; two bile acids, glycocholic acid (GCA) and glycodeoxycholic acid, (GCDA); two vitamin E metabolites (2,5,7,8-tetramethyl-2-(2’-carboxyethyl)-6-hydroxychroman (α-CEHC) and 7,8-Trimethyl-2-(beta-carboxyethyl)-6-hydroxychroman (γ- CEHC): a food additive (Naringenin) and plant compound (salicylate). Salicylate occurs widely in fruits and vegetables as well as aspirin

## Methods and materials

### Subjects

The study was approved by the Institutional Review Boards of Rutgers University-New Jersey Medical School, Rowan University-School of Osteopathic Medicine and their predecessor, the University of Medicine and Dentistry of New Jersey (UMDNJ). Subjects were recruited from the Pediatric Neurology and Pediatrics clinical practices of Rutgers-New Jersey Medical School. Written informed consent was obtained from each child’s guardian or parent as appropriate by either an investigator (XM) or a team member. A member of the clinic staff witnessed the procedure.

The study population consisted of three groups of children from 3–16 years old. All the children who were diagnosed with ASD were under the care of a pediatric neurologist (X.M), and the diagnoses were made according to the Diagnostic Statistical Manual IV-TR and/or V, and were further confirmed by Autism Diagnostic Interview-Revised, Autism Diagnostic Observation Scale-Generic, or both rating scales. Medical histories and comorbidity data were collected for the ASD and ADHD subjects. The diagnosis of ADHD was also made according to DSM-IV-TR criteria and DSM V and confirmed with the Vanderbilt ADHD diagnostic scale. Although DSM V was released in 2013, the DSM-IV-TR criteria for ASD and ADHD continued to be used to ensure that the diagnostic criteria would be the same as those employed in our earlier BPA and DEHP studies [30, 39, 40]. Healthy children compromised a third group, the control group were recruited from the Pediatric Ambulatory Care Center of our Institute. Efforts were made to age match the three groups. Spot urine specimens were collected from each child between 10:00 a.m. and 4:00 p.m. [39]. The samples were frozen and then stored at −70°C within 2 hours of collection.

Because medical and psychiatric comorbidities along with intellectual impairment are frequent among ASD and ADHD children [41], we included ASD and ADHD children who were diagnosed with or without comorbid disorders. There were no exclusionary criteria with respect to levels of intellectual performance. However, children with ASD and ADHD and a known genetic disorder, e.g., Fragile X syndrome, were excluded. The children in the control group were screened for medical and developmental disorders in addition to chart reviews, and only children without chronic or recurrent medical disorders were considered healthy and included in the study. The subjects were carefully screened for signs of infection or other illnesses on the day of specimen acquisition, and subjects with acute illness were excluded. The dietary intake history was recorded within 24 hours of the urine collection and included current medications being taken and vitamin intake.

Analytical methodology. The urinary creatinine concentrations were measured by us using a kit (Sigma Aldrich, St. Louis, MO 68178), and by LC-MSMS by Metabolon as part of the Metabolome. Metabolite concentrations measured by us were normalized to the Sigma-Aldrich kit values and the metabolome determined metabolites to the metabolome creatinine values. The reason for using metabolome creatinine values for metabolome metabolites was that the metabolome values correct for the possibility of any evaporative losses during sample preparation (aliquoting) for Metabolon. The correlation by regression analysis between the two methods was r^2^<0.95.

The analytical methodology for measuring BPA and Phthalates in urine has been previously described [30, 40]. Briefly, the concentration of free and total BPA in the collected urine specimens was measured by isotope dilution-liquid chromatography mass spectrometry–mass spectrometry (ID-LC-MSMS) using the methodology Koch et al for phthalates and our modifications of the BPA methodology of Liao and Kannan for BPA [22, 25, 30, 42]. The limits of detection for phthalates was 0.5 to 1ng/ml for phthalates [25] and 0.2 ng/ml for BPA [30]. The ID-LC-MSMS assays measure free BPA and free phthalates. To measure the total BPA or phthalate present in the urine, the glucuronidated plasticizers has to be deconjugated by treating the urine with β-glucuronidase to remove the glucuronic acid residue from the glucuronidated BPA or phthalate (Sigma-Aldrich, St. Louis MO).

### Metabolomic analyses

Urine specimens were sent to Metabolon, Inc. (Morrisville, NC 27560) for metabolomic analysis. The results were used for two purposes. Firstly, for calculating the efficiency of glucuronidation (= % bound) for certain compounds in the metabolome and secondly for analysis of the distribution of compounds in the metabolome.

The requirements for being able to calculate a value for % bound from the metabolome is the presence of both free and bound compounds in the metabolome, total absence of bound compound after treatment of the urine with glucuronidase and a corresponding increase in the amount of free compound in the metabolome. The metabolomic screen gives values for the area-under-the -curve data (AUC) for the free and bound compounds. The measured area is a function of the amount of compound present, the fragmentation pattern and the mass spectrometer settings. The AUC cannot be used directly for calculating the efficiency of glucuronidation (% bound) because the fragmentation patterns for the free and bound compound are different. To calculate the total amount of a compound present and bound, it is necessary to first convert glucuronide areas into their free compound equivalents. The necessary conversion factors were determined by selecting 20 random urine specimens, preparing two aliquots, ‘control’ and ‘treated’. A fresh solution containing ~250–300 units/ml of E. Coli β-glucuronidase in 1M Ammonium Acetate was prepared immediately before use. Either 1 ml of enzyme solution (treated) or 1 ml of 1M ammonium acetate (control) was added to the control and treated tubes respectively. The tubes were incubated at 37°overnight (~19 hours) after which an aliquot was removed, frozen and sent to Metabolon, Inc., (Morrisville, NC 27560) for metabolomic analysis.

The amount of glucuronide hydrolyzed to free compound was obtained from the treated metabolome and a conversion factor for glucuronide to free compound equivalents. The control urine gives the AUCs for free (F) and, separately, glucuronidated (G) compound in the urine. The enzyme treated group gave a value for the total amount of compound T, (F + G) present in the original sample. The glucuronidated area was converted to free compound area equivalents by multiplying it by the conversion factor (R). The % free metabolite was calculated using the equation: % free = 100*(F/(T*R)). The % bound (efficiency of glucuronidation) values for the 12 glucuronidation metabolites were then calculated by subtracting the respective % free metabolite value from 100.

Statistical Analyses. In calculating the frequency distributions for the % bound values, two subjects were found to have negative % bound values for BPA suggesting one of the two parameters (F or G) used for the calculation was in error. Because negative percentages for % bound metabolites are impossible, the data for the two children with negative percentages were excluded from further analyses involving BPA calculations but were included in analyses involving the other 11 glucuronides. In addition, compounds were excluded if the number of detections for a particular metabolite was < 50% of the sum of the total number of metabolites found in the ASD, CTR, or ADHD groups. No missing data were imputed.

The metabolome metabolites were normalized child’s urinary creatinine concentration [35, 43]. An alternate way would have been to control for creatinine. For the present study, normalization of the metabolome data to creatinine is the preferred way because creatinine is being used to give a value for excretion over time [43, 44]. In our prior BPA study, the BPA metabolite values were controlled for by regressing the BPA values on the child’s gender, ethnicity, age, and creatinine levels after applying log10 transformations to decrease the severity of skewness [30]. However, we found that the latter parametric approach yielded results that were comparable to just employing nonparametric statistics to compare mean ranks of the metabolites among groups, such as Kruskal-Wallis H test and calculating Spearman rank-order correlations (r_s_), instead of Pearson product-moment correlations (r_p_) to estimate the magnitudes of the relationships of the BPA indices with respect to other types of metabolites. The use of r_s_ is a valid approach to for estimating the magnitudes of association when data are minimally to moderately skewed and there are only several extreme outliers [45]. The statistical analyses were performed with either EXCEL 365 or SPSS29.

## Results

The raw data, from which all that follows has been derived is given in the S1 Dataset. Table 1 displays the characteristics of the three samples. The percentages of boys in each group are listed along with the mean ages, BMI levels, and urinary creatinine levels for each of the three groups. None of these four characteristics significantly differentiated among the three groups after applying a Bonferroni adjustment of α of 0.05/4 = 0.0125, two-tailed test of significance to control for the familywise error rate of performing four comparative analyses. Therefore, we concluded that gender, age, and BMI did not have to be controlled for in comparing the three groups’ metabolite levels.

**Table 1:** Subject characteristics.

CHARACTERISTIC	GROUP	*N*	*%*	STATISTIC	*P*	EFFECT SIZE	
Sex (boys)	ASD	66	74	χ2 (2) = 5.24	0.07	Cramer’s V = .19	
	CTR	37	54				
	ADHD	46	74				
		N	M	SD			
Age (years)	ASD	65	10.11	3.41	*F* (2,144) = 3.32	0.04	η2 = .04
	CTR	37	8.78	2.96			
	ADHD	45	10.53	3.00			
BMI	ASD	63	21.87	6.57	*F* (2, 138) = 1.36	0.26	η2 = .02
	CTR	33	19.82	4.62			
	ADHD	45	20.89	5.63			
Creatinine (mg/ml)	ASD	66	1.08	0.64	*F* (2, 146) = 1.78	0.17	η2 = .02
	CTR	37	1.31	0.58			
	ADHD	46	1.29	0.89			

Table 2 presents the mean, median, geometric mean, min-max, and % of children with data for each analyte by group. After making the necessary Bonferroni adjustments, none of the data is significant at the p<0.05 level.

**Table 2:** a. free compounds. b. total compounds.

a											
PARAMETER	MEAN (SD)				MEDIAN			GEOMETRIC MEAN			
5 OXO-MEHP	0.26 (0.38)	0.14 (0.17)		0.22 (0.21)	0.15	0.09	0.15	0.14	0.08		0.14
5 CX-MEPP	41 (143)	11 (14)		17 (17)	9	7	10	11	6		12
5 OH-MEHP	0.67 (1.51)	0.33 (0.38)		0.57 (0.60)	0.33	0.26	0.39	0.29	0.19		0.36
MEHP	2.11 (5.95)	0.56 (0.38)		0.89 (1.42)	0.60	0.47	0.54	0.73	0.47		0.61
BPA	1.33 (4.21)	0.45 (0.42)		0.44 (0.41)	0.35	0.37	0.27	0.43	0.35		0.32
α-CEHC	108 (105)	115 (169)		86 (80)	79	66	63	79	74		63
γ-CEHC.	256 (239)	222 (262)		344 (313)	178	155	226	177	146		246
CORTISOL.	71 (99)	87 (111)		39 (24)	44	48	33	47	55		32
GLYCODEOXYCHOLATE	93 (92)	68 (47)		101 (76)	66	55	88	66	53		75
GLYCOCHOLATE	383 (403)	257 (228)		389 (309)	233	202	315	255	185		290
NARINGENIN	58 (71)	43 (59)		34 (33)	27	17	18	29	23		22
SALICYLATE	2128 (3661)	4158 (18164)		2287 (2813)	1164	848	1114	1151	978		1290
PARAMETER	MINIMUM VALUE				MAXIMUM VALUE			% WITH DATA			
5 OXO-MEHP	0.02		0.02	0.02	3	1	1	85		98	95
5 CX-MEPP	2		1	3	949	66	75	91		100	100
5 OH-MEHP	0.01		0.01	0.04	11	2	3	87		98	97
MEHP	0.16		0.09	0.20	35	2	9	91		100	100
BPA	0.04		0.05	0.09	28	2	2	88		100	100
α-CEHC	19		27	7	511	959	379	66		71	68
γ-CEHC.	23		27	36	1314	1629	1398	96		100	100
CORTISOL.	8		10	8	665	479	98	82		84	84
GLYCODEOXYCHOLATE	13		6	7	520	212	312	91		98	97
GLYCOCHOLATE	38		17	26	1968	1089	1614	99		100	100
NARINGENIN	2		2	6	276	262	128	46		67	50
SALICYLATE	187		151	175	25976	125854	13704	99		100	100
b											
PARAMETER	MEAN (SD)				MEDIAN			GEOMETRIC MEAN			
5 OXO-MEHP	18 (57)		12 (12)	7 (8)	6	7	4	7		8	4
5 CX-MEPP	85 (311)		33 (32)	21 (22)	18	22	14	22		24	13
5 OH-MEHP	29 (100)		19 (20)	11 (12)	9	12	8	10		12	7
MEHP	49 (9)		4 (3)	2 (3)	2	3	1	3		3	2
BPA	12 (69)		7 (21)	2 (1)	2	3	1	2		3	1
α-CEHC	1440 (1186)		1198 (837)	1249 (1605)	913	971	804	1063		973	891
γ-CEHC.	113 (138)		70 (41)	124 (131)	81	63	77	80		57	85
CORTISOL.	8844 (6414)		9542 (5078)	8427 (6255)	6989	9026	7092	7174		8142	6643
GLYCODEOXYCHOLATE	390 (260)		298 (164)	251 (159)	311	269	191	308		250	205
GLYCOCHOLATE	485 (434)		450 (315)	305 (242)	319	353	264	356		363	235
NARINGENIN	5316 (7329)		6116 (8088)	2663 (2853)	2079	1716	1065	2052		2463	1238
SALICYLATE	3246 (4149)		3271 (3269)	10370 (51838)	2191	1887	1704	2232		2176	1908
PARAMETER	MINIMUM VALUE				MAXIMUM VALUE			% WITH DATA			
5 OXO-MEHP	0		2	1	441	61	32	91		100	100
5 CX-MEPP	2		7	3	2112	146	96	91		100	100
5 OH-MEHP	1		1	1	785	106	53	91		100	100
MEHP	0		1	0	52	19	15	91		100	100
BPA	0		1	0	530	129	6	88		100	100
α-CEHC	173		143	184	5197	4112	7587	99		100	98
γ-CEHC.	8		7	9	1028	163	619	94		95	96
CORTISOL.	1705		1318	1596	35951	24245	35381	96		100	98
GLYCODEOXYCHOLATE	48		51	27	1281	656	651	82		97	96
GLYCOCHOLATE	73		98	37	2044	1657	1208	79		95	91
NARINGENIN	119		107	121	30748	26278	8480	61		39	62
SALICYLATE	468		401	266	29197	14537	349510	99		100	100

Analytical data for plasticizers. Table 2A gives the mean, median, geometric mean, min-max, and % of children with data for each analyte by group for the free compound, Table 2B for the total compound (free plus glucuronidated). Note that absence of data could imply below the limits of detection or poorly resolved mass spectrometer peak and therefore unable to obtain a measurement. For the five plasticizers the units are ng/mg creatinine. For the other 7 compounds where the data was derived from the metabolome, the units are LC-MSMS chromatogram area (raw data from Metabolon) normalized to creatinine and divided by 10^6^. The division by 10^6^ is solely for readability.

Twelve sets of % glucuronidation values were available for data analyses. Five were plasticizers, BPA the primary metabolite of DEHP, MEHP plus three of its secondary metabolites, 5 OXO-MEHP, 5 CX-MEPP and 5 OH-MEHP. MEHP is a more potent endocrine disruptor than the parent compound or its metabolites [46, 47]. None of these were detected in the metabolome. Their concentrations were below the detection threshold of the metabolome methodology used.

The other seven glucuronidation efficiencies were derived from metabolites present in the metabolome. The criteria for being useable were (i) both the compound (free) and its glucuronide (bound) were present in the metabolome, (ii) after treatment with glucuronidase no glucuronide was present in metabolome, (iii) there was a parallel increase in the amount of free compound present, (iv) % bound values (= efficiency of glucuronidation) had to be obtained for at least half the subjects and (iv) the glucuronidation efficiencies covered a reasonable range (Table 2).

Seven metabolome compounds met all of these criteria. There was one steroid (cortisol), two bile acids (glycocholic acid (GCA) and glycodeoxycholic acid, (GCDA)), two vitamin E metabolites (2,5,7,8-tetramethyl-2-(2’-carboxyethyl)-6-hydroxychroman (α-CEHC) and 7,8-Trimethyl-2-(beta-carboxyethyl)-6-hydroxychroman, (γ- CEHC)), and two plant compounds, the fruit flavonoid Naringenin and Salicylate. Salicylate occurs widely in fruits and vegetables as well as the drug aspirin. When drugs are the source, Salicylate level in the urine are orders of magnitude greater than the background level. There was one such instance in the present study; a subject was found to have a level approximately six times higher than the total sample’s Salicylate mean. This data point was dropped from all further analyses.

The quantification of the data and subsequent statistical analyses were conducted in stages. First, the skewness indices of the frequency distributions for the 12 glucuronidation efficiencies (% bound) were calculated. There were five metabolites with negative skewness indices < -1.0 and one metabolite with a positive skewness > 1.0. Therefore, we used nonparametric statistical analyses for comparisons between the 12 glucuronidation efficiency data sets.

To determine whether there was sufficient variability within each of the 12 glucuronidation pathways to support further analyses, the frequency distribution for each of the 12 metabolites was divided into tertiles, highest, middle and lowest. The means and standard deviations shown in Table 2 for each of the 12 metabolites’ tertiles indicated that there was sufficient spread in the % bound glucuronidation levels to support further analyses.

Metabolon reports the metabolome results with each detected compound categorized into super-families and sub-pathways using Metabolon’s classification scheme. There were 8 super-pathways, Amino Acids (n = 226), Carbohydrates (n = 29), Cofactors and Vitamins (n = 23), Energy (n = 14), Lipids (n = 124), Nucleotides (n = 53), Peptides (n = 27), Xenobiotics (142) and unclassified compounds (n = 44). The super-pathways are broken up into numerous sub-pathways. Examples from the Amino Acids super-pathway are Glutamate metabolism, Histidine metabolism. Leucine, Isoleucine and Valine Metabolism etc. The Xenobiotics super-family include Drugs, Food additives, Chemicals etc. The two largest super-pathways were Amino Acids and Xenobiotics.

Analyses of mean fold differences between the means of the metabolites in the metabolome were used to confirm that the three groups of children were metabolically distinct and that correlational analyses to confirm associations among the plasticizers and clinical state were statistically warranted. To demonstrate that the three groups of children were metabolically different, the mean fold ratios of the means for the ASD, CTR, and ADHD groups were calculated for each of the metabolites in the metabolome (Table 3). The standard deviations (SD) of the Xenobiotic super-family, which encompassed a mixture of drugs, food additives, and various environmental chemicals were greater than for the other metabolite groupings (Table 4).

**Table 3:** Range of glucuronidation efficiencies.

TERTILE/ PATHWAY	HIGHEST	MIDDLE	LOWEST
	MEAN (SD)	MEAN (SD)	MEAN (SD)
5 OXO-MEHP (N = 143)	95.22 (4.62)	98.28(0.33)	99.24 (0.40)
5 CX-MEPP (N = 138)	34.6 (10.06)	51.87 (2.98)	65.29 (6.63)
5 OH-MEHP (N = 142)	93.51 (5.55)	97.44 (0.35)	98.81 (0.62)
MEHP (N = 142)	39.45 (9.95)	69.04 (6.98)	87.47 (4.84)
BPA (N = 140)	51.78(17.63)	79.08 (4.52)	91.95 (3.76)
α-CEHC (N = 148)	88.72 (3.59)	95.98 (1.41)	100 (0)
γ-CEHC (N = 146)	95.25 (2.48)	98.00 (0)	99.07 (0.25)
CORTISOL (N = 124))	31.39 (7.53)	48.9 (3.85)	66.61 (9.81)
GLYCHODEOXYCHOLATE (N = 134)	54.88 (9.19)	73.35 (3.28)	85.75 (4.65)
GLYCHOCOLATE (N = 128)	3.1 (1.25)	9.11 (2.22)	30.07 (17.63)
NARINGENIN (N = 81)	95 (6.04)	99.35 (0)	100 (0)
SALYCYLATE (N = 148)	19.72 (6.87)	42.22 (6.90)	66.76 (8.19)

Range of Glucuronidation Efficiencies (= % bound) for the 12 glucuronidation pathways by tertile. Abbreviations: Mono-2-ethylhexyl phthalate (MEHP), mono-(2-ethyl-5-oxohexyl) phthalate (5-oxo MEHP), mono-(2-ethyl-5-carboxypentyl phthalate (5-CX MEPP), mono-(2-ethyl-5-hydroxyhexyl) phthalate (5-OH MEHP), BPA Bisphenol A; α-CEHC, γ-CEHC, GlyCO, Glycocholate; Glydeox, Glycodeoxycholate.

**Table 4:** Fold ranges.

CLASS OF METABOLITES	N	ASD / CTR	SD	ASD / ADHD	SD	ADHD / CTR	SD
Total Metabolome	692	1.08*	0.53	1.18**	0.79	0.99	0.53
Amino acids	193	1.01	0.28	1.12**	0.28	0.92**	0.14
Non-essential amino acids	41	1.07	0.32	1.14**	0.32	0.95	0.13
Essential amino acids	152	1.00	0.25	1.11**	0.25	0.91**	0.12
Carbohydrates	43	1.04	0.26	1.19	0.26	0.89	0.13
Vitamins and Co-factors	33	1.04	0.17	1.20*	0.29	0.89*	0.17
Fatty acids	52	1.06	0.45	1.14	0.39	0.97	0.25
Steroids	26	1.51*	0.61	1.05	0.25	1.46**	0.51
Phospholipids	19	1.18	0.38	1.26	0.34	0.93	0.12
Bile acids	21	0.98	0.23	1.24*	0.27	0.82	0.23
Purines and pyrimidines	53	0.94	0.15	1.14	0.29	0.84	0.29
Xenobiotics	142	1.19	0.95	1.16	0.33	1.15	0.84
All other compounds	110	1.11	0.49	1.16	0,33*	1.04	0.46

Mean Fold Difference Ratios by Type of Group for Different Classes of Metabolites. *p<0.05, **p<0.01.

Examination of the 151 metabolites in the Xenobiotic super-family showed that this was attributable to highly skewed data for two drugs, Acetaminophen and Ibuprofen, and the artificial sweeteners, Sucralose, Acesulfame K and Saccharine and their metabolites. This observation is not surprising because these drugs are common analgesics, and the three sweeteners are frequently added to low calorie drinks. Using z score analyses, number of SDs above the cluster’s mean, to identify outliers for these five compounds, we found z scores > 10 and even > 25. Eliminating these five compounds and their metabolites from the Xenobiotics cluster reduced the N from 151 to 142 for Xenobiotics and brought the SDs into the same range as the other compound families in the Metabolome. Removing them from the total metabolome (n = 701→n = 692) had only minimal effect on the statistical analyses because of the large number of compounds in the metabolome. A few steroid values appeared to be appeared to be anomalously high (<2SD from mean) across all groups. The likely cause was androgens in the steroid cluster (7 out of 22 compounds); there is a higher percentage of boys in the ASD and ADHD groups, hence more androgens [48].

The frequency distributions of mean fold differences were then graphically examined and approached those that might be expected for a normal distribution, and independent t tests were considered appropriate for estimating the significance of mean differences among the groups. However, some of the significant mean ratio differences between groups disappeared after application of a Bonferroni correction.

Some differences remained. For the total Metabolome, with or without inclusion of analgesics and soda sweeteners, the mean fold ratios were statistically significant for the ASD/CTR and ADHD/CTR mean ratios indicating that both the ASD and ADHD mean fold ratios were different from each other and the controls. There also were significant differences for the amino acids and vitamins-co-factors super-pathways. Mean fold ratios for the ASD and ADHD groups differed significantly from the CTR group, but not from each other. For the purposes of this study, the need was to establish that the three groups of children were metabolically distinct, and the mean fold ratio data that are displayed in Table 3 support of such differentiation.

Table 5 shows the Spearman correlations (r_s_s) among the 12% bound glucuronidation pathways. As expected for closely related metabolic groups, there are multiple significant correlations. Although the pattern of correlations is complex, it is not random. The phthalates are chemically related, and their r_s_s tend to cluster together. The two bile acids correlate only with each other. Cortisol seems to be unique as does Naringenin. Details about the relationships among putative groupings of the 12 glucuronidation efficiencies are tangential for the present study; the requirement for the present study is that the 12 metabolites’ r_s_s display different patterns of significant relationships indicating that although the pathways are related, they do differ (Table 5).

**Table 5:** Spearman correlations.

	5 OXO-MEHP	5 CX-MEPP	5 OH-MEHP	MEHP	BPA	CORTI-SOL	α-CEHC	γ-CEHC	GLYCO	GLYCO-DEOX	NARIN-GENIN	SALICY-LATE
5 OXO-MEHP	--	0.076	.607**	0.100	-0.048	0.072	0.132	.467**	-0.142	-0.029	0.175	.363**
5 CX-MEPP	0.076	--	.194*	-.263**	-.183*	-0.083	.272**	.299**	-0.042	0.018	-0.221	.232**
5 OH-MEHP	.607**	.194*	--	-0.064	-0.129	0.103	.200*	.505**	-0.087	0.045	-0.023	.563**
MEHP	0.100	-.263	-0.064	--		0.162	-0.124	-.239**	-0.027	-0.020	0.134	-0.022
BPA	-0.048	-.183*	-0.129	.167**	--	.302**	.249**	-0.164	0.022	0.133	0.067	0.003
α-CEHC	0.132	.272**	.200*	-0.124	.249**	--	-0.047	.383**	-0.105	-0.091	-.225*	0.109
γ-CEHC	.467**	.299**	.505**	-.239**	-0.164	-0.050	.383**	--	-0.118	0.022	-0.206	.538**
CORTISOL	0.072	-0.083	0.103	0.162	.302**	-0.047	--	-0.050	0.014	0.177	0.054	.281**
GLYCO	-0.142	-0.042	-0.087	-0.027	0.022	0.014	-0.105	-0.118	--	.681**	0.048	-0.115
GLYDEOX	-0.029	0.018	0.045	-0.020	0.133	0.177	-0.091	0.022	.681**	--	0.169	0.053
NARINGENIN	0.175	-0.221	-0.023	0.134	0.067	0.054	-.225*	-0.206	0.048	0.169	--	-.296**
SALYCYLATE	.363**	.232**	.563**	-0.022	0.003	.281**	0.109	.538**	-0.115	0.053	-.296**	--

**. Correlation significant at the 0.01 level (2-tailed).

*. Correlation significant at the 0.05 level (2-tailed).

Spearman correlations coefficients between the 12 glucuronide conjugation. Abbreviations: Mono-2-ethylhexyl phthalate (MEHP), mono-(2-ethyl-5-oxohexyl) phthalate (5-oxo MEHP), mono-(2-ethyl-5-carboxypentyl phthalate (5-CX MEPP), mono-(2-ethyl-5-hydroxyhexyl) phthalate (5-OH MEHP), BPA Bisphenol A; α-CEHC, γ-CEHC, GlyCO, Glycocholate; Glydeox, Glycodeoxycholate. efficiencies.

*p<0.05

** p<0.01.

Table 6 displays the nonparametric comparisons of the mean ranks among the ASD, CTR, and ADHD groups for the 12 glucuronidation % bound metabolites using Kruskal-Wallis H tests. A Bonferroni adjustment of 0.05/12 (= 0.0125) was employed to control for the familywise error rate from conducting 12 mean rank comparisons. The BPA pathway was the only pathway that significantly discriminated among three groups of children (p<0.001). There was a trend for a similar relationship with MEHP (p<0.03), but after the Bonferroni adjustment, the Kruskal-Wallis H statistic was not significant.

**Table 6:** a. Glucuronidation pathway analysis. b. Comparisons.

a									
PATHWAY	N-ASD	ASD MEAN RANK	N-CTR	CTR MEAN RANK	N-ADHD	ADHD MEAN RANK	K-W H (1)	P	η2
% Bound 5 OXO-MEHP	60	70.35	37	78.50	46	68.92	1.26	0.53	0.01
% Bound 5 CX-MEPP	57	66.76	35	71.06	46	71.71	0.46	0.79	0.01
% Bound 5 OH-MEHP	60	71.88	36	73.08	46	69.77	0.14	0.93	0.01
% Bound MEHP	60	68.38	36	87.08	46	63.38	7.30	0.03	0.04
% Bound BPA	58	68.99	37	89.45	45	56.87	13.24	<0.001	0.08
% Bound α-CEHC	66	76.52	37	74.64	45	71.42	0.40	0.82	0.01
% Bound γ-CEHC	64	75.88	37	61.43	45	80.03	4.50	0.10	0.02
% Bound Cortisol	54	65.06	31	71.11	39	52.12	5.31	0.07	0.03
% Bound Glycocholate	55	72.42	36	57.51	43	69.57	3.39	0.18	0.01
% Bound Deoxyglycocholate	53	66.51	34	63.53	41	62.71	0.28	0.87	0.01
% Bound Naringenin	40	43.54	14	44.14	27	35.61	2.25	0.32	0.0
% Bound Salicylate	65	78.14	37	65.24	46	76.80	2.33	0.32	0.0
b									
PLANNED CONTRASTS, % BOUND BPA	*U*	*P*	*d*						
ASD < CTR	784	0.03	0.47						
ASD > ADHD	981	0.03	0.51						
ADHD < CTR	396	<0.001	1.00						
POST HOC COMPARISONS FOR % BOUND MEHP									
ASD = CTR	891	0.15	0.29						
ASD = ADHD	1,003	0.16	0.40						
ADHD < CTR	484	<0.001	0.77						

Comparisons of the 12 glucuronidation pathways among ASD, CTR, and ADHD Groups by Kruskal-Wallis H Tests with a Bonferroni adjustment for alpha/12 (= 0.0125) to control for the familywise error rate (Table 6A) followed up by Mann-Whitney U Tests with *a prior*i planned contrasts for % Bound BPA and Bonferroni post-hoc comparisons for % Bound MEHP (Table 6B).

The mean ± SEM values for glucuronidation efficiency of the BPA pathway were ASD 72.8 ± 2.9%, CTR 69.3 ± 2.7% and ADHD 69.3 ± 2.7%. Compared to the control group, the reductions for the ASD and ADHD groups were statistically significant. Expressed as percentages, the reductions in glucuronidation efficiency are 11% for the ASD (p = 0.020) and 15% for the ADHD (p<0.001) groups. MEHP showed similar, but non-significant trends; ASD 57.6 ± 4.3%, CTR 69.2 ± 3.4% and ADHD 61.7 ± 4.3%) corresponding to reductions of the efficiency of glucuronidation of 17% for ASD and 11% for ADHD.

The next step in the statistical analyses was to examine the relationships between the metabolome metabolites and the 12 pathways with each clinical state (ASD, CTR and ADHD). First the r_s_s were calculated to determine what the relationships of the % bound values were for each of the 12 pathways with each of the 692 metabolites in the metabolome. Only the number of positive and negative r_s_s that were significant at ≥ 0.05 level, two-tailed test, were included in the subsequent analyses. No attempt was made to control for the plausibly high false positive rate that would occur from calculating 478,864 (3 x 12 x 692) r_s_s. Our focus was on identifying chemically recognizable patterns of relationships. Accordingly, we examined the results by familial groupings.

**Fig 1:**
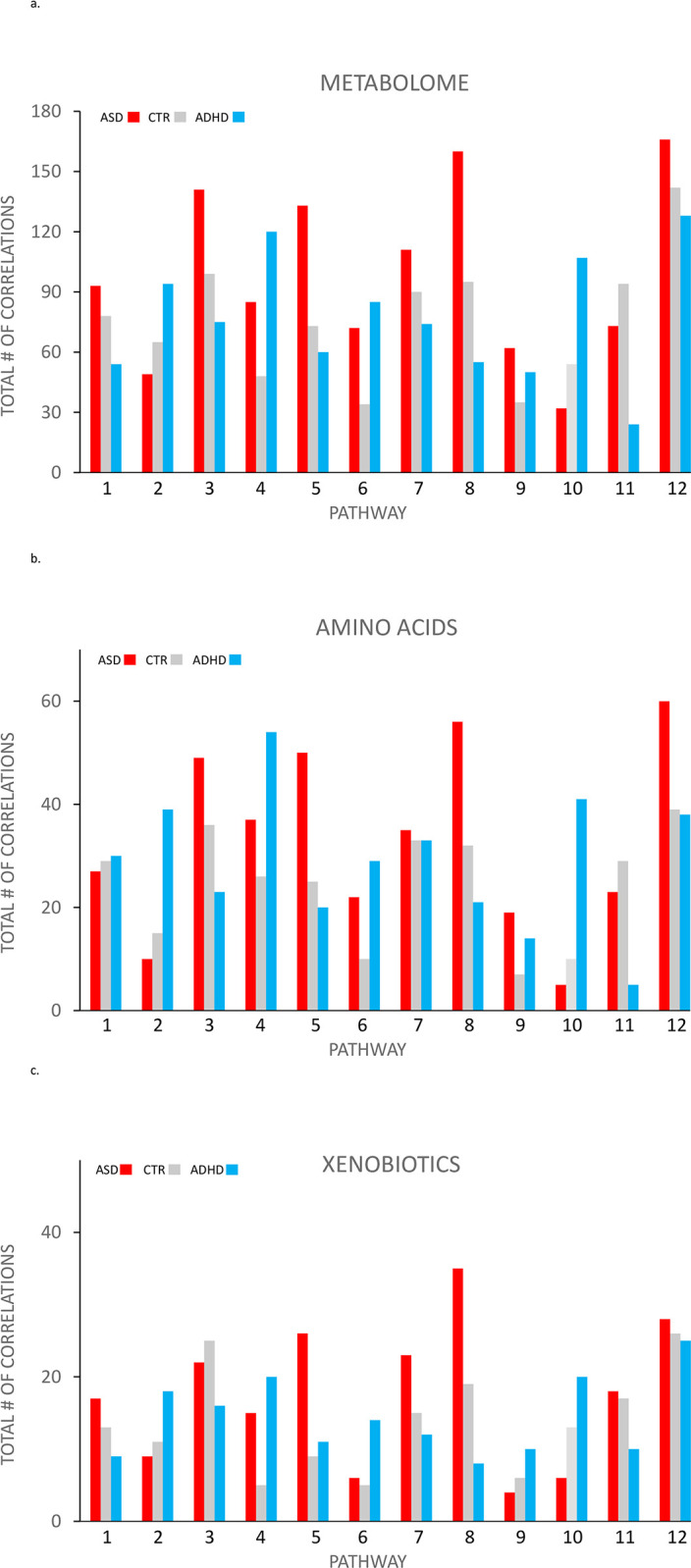
a, b and c. Relationship between the total number of statistically significant correlations for each of the 12 glucuronidation pathways with ASD, CTR and ADHD for the 692 compounds in the metabolome (Fig 1a), amino acids (Fig 1c) and Xenobiotic super-families. 1 = 5-OXO MEHP, 2 = 5-X MEHP, 3 = 5-OH 4 = MEHP, 5 = BPA, 6 = GLYCOCHOLATE, 7 = GLYCODEOXYCHOLATE, 8 = CORTISOL, 9 = α-CEHC, 10 = γ-CEHC, 11 = NARINGNIN, 12 = SALYCYLATE.

Fig 1A plots the total number of statistically significant sign independent Spearman correlations between the Metabolome constituents (n = 692) and each of the 12 glucuronidation pathways for the ASD, CTR and ADHD groups. Similar patterns are found when two other ‘super-families’ amino acids (Fig 1B) and xenobiotics (Fig 1C) are plotted separately. The pattern is varied and complex, but there is nothing obviously apparent that relates to clinical status. The complex pattern of results is consistent with the 12 glucuronidation pathway being related, but not identical.

**Fig 2:**
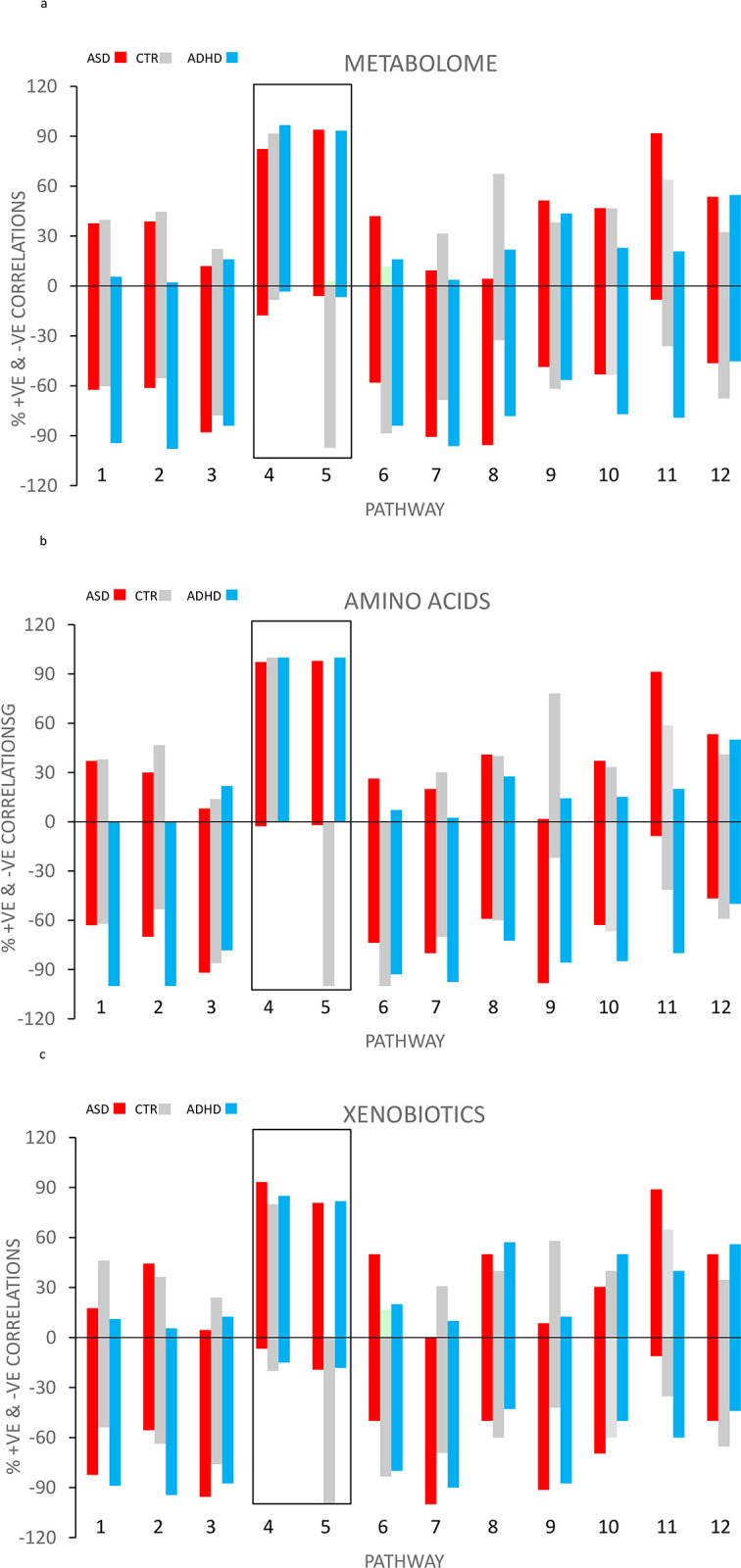
a. % of total number Spearman correlations from Fig 1A–1C either positive or negative by glucuronidation pathway for the total Metabolome (Fig 2a), amino acids (Fig 1C) and Xenobiotic super-families. 1 = 5-OXO MEHP, 2 = 5-X MEHP, 3 = 5-OH 4 = MEHP, 5 = BPA, 6 = GLYCOCHOLATE, 7 = GLYCODEOXYCHOLATE, 8 = CORTISOL, 9 = α-CEHC, 10 = γ-CEHC, 11 = NARINGNIN, 12 = SALYCYLATE.

The situation changes when the distributions of positive and negative correlations are examined as percentages of the total number of significant (*p* ≤ .05) correlations. The sum of the % positive and % negative correlations equals 100%. Fig 2A is for the total Metabolome (n = 692), whereas Fig 2B is for the Amino Acids super-pathway (n = 226) and Fig 2C is for Xenobiotics (n = 141). The three figures are similar. The numbers are not large enough to obtain meaningful plots for the other super-families, but if they are combined a similar plot to those shown in Fig 2A–2C is obtained. Marked differences between ASD, CTR and ADHD are now found.

Although the three figures are very similar, the differences between ASD, CTR and ADHD are most clearly seen in the amino acids plot. In contrast to the 9 of the other 10 pathways, virtually no negative correlations were found with ASD and ADHD for the BPA and MEHP pathways. Nearly all the correlations were positive for ASD and ADHD with BPA and MEHP.

However, ASD and ADHD differed in their relation to the control group. For the MEHP pathway there was no difference from the control group for either negative or positive correlations (Fig 2A–2C). For the BPA pathway the control group showed almost exclusively negative correlations in contrast to the MEHP pathway which showed a preponderance of positive control correlations. The figures confirm that the BPA and MEHP pathways are similar in some ways and different in other ways and both are different from the other 10 glucuronidation pathways.

We then examined the metabolome to determine whether any of the individual positive and negative correlations were common between the groups of children. ASD and ADHD had the most in common had the most in common (n = 23) and the control group the least, 13 for CTR and ASD, 4 for CTR vs ADHD. Of the 23 in common between ASD and ADHD, the majority were with the amino acids grouping (Table 7). This is further evident that at the metabolic level, the three groups of children are different but the two groups of children with neurodevelopmental disorders have commonalities not shared with healthy control children.

**Table 7:** Commonality.

SUPER-PATHWAY	PATHWAY	COMPOUND NAME
Amino Acid	Alanine and Aspartate Metabolism	N-acetylalanine
Amino Acid	Glutathione Metabolism	5-oxoproline
Amino Acid	Histidine Metabolism	1-methylhistamine
Amino Acid	Leucine, Isoleucine and Valine Metabolism	2-methylbutyrylglycine
Amino Acid	Leucine, Isoleucine and Valine Metabolism	isoleucine
Amino Acid	Leucine, Isoleucine and Valine Metabolism	methylsuccinate
Amino Acid	Leucine, Isoleucine and Valine Metabolism	N-acetylleucine
Amino Acid	Lysine Metabolism	N2,N6-diacetyllysine
Amino Acid	Polyamine Metabolism	(N(1) + N(8))-acetylspermidine
Amino Acid	Polyamine Metabolism	N1,N12-diacetylspermine
Amino Acid	Tryptophan Metabolism	C-glycosyltryptophan
Amino Acid	Tyrosine Metabolism	homovanillate (HVA)
Amino Acid	Tyrosine Metabolism	vanillactate
Carbohydrate	Pentose Metabolism	arabitol/xylitol
Lipid	Fatty Acid Metabolism(Acyl Glycine)	2-butenoylglycine
Lipid	Fatty Acid, Dicarboxylate	adipate (C6-DC)
Lipid	Fatty Acid, Dicarboxylate	azelate (C9-DC)
Lipid	Fatty Acid, Dicarboxylate	pimelate (C7-DC)
Lipid	Fatty Acid, Dicarboxylate	suberate (C8-DC)
Nucleotide	Purine Metabolism, Adenine containing	N6-succinyladenosine
Nucleotide	Purine Metabolism, Guanine containing	2’-deoxyguanosine
Nucleotide	Pyrimidine Metabolism, Uracil containing	N3-methyluridine
Xenobiotics	Food Component/Plant	erythritol

Detailed list of all compounds correlating with both ASD and ADHD

## Discussion

There is an extensive body of epidemiological evidence for a relationship between neurodevelopmental disorders and environmental pollutants such as plasticizers [34, 38]. Beyond showing that plasticizers function as wide-acting endocrine disruptors there is little data on the metabolic processes linking plasticizer exposure to neurodevelopmental disease in humans. Ultimately the effects of both genetics and Xenobiotics are expressed through metabolic processes.

In the present study, two semi-independent approaches were used for analyzing the data to demonstrate a linkage between plasticizer metabolism and neurodevelopmental disorders. We use the term semi-independent rather than independent because both approaches share a dependence on the value for glucuronidation efficiency for the 12 pathways. Approach #1 uses only the glucuronidation efficiency data; approach #2 uses the interaction between the 12 pathways and the metabolome. Genetic factors are likely to be important factors in determining the efficiency of glucuronidation.

Table 6 summarizes results of the first approach. The table examines the relationship of the efficiency of the 12 glucuronidation pathways to ASD and ADHD. As expected from our earlier study [30], ASD showed a correlation to a decrease in BPA glucuronidation efficiency. Unexpectedly the ADHD group also showed a correlation with the BPA pathway. The control group showed no relationship to any of the pathways. The two neurodevelopmental disorders shared a common association with a decreased ability to detoxify BPA.

Like the BPA pathway, the MEHP pathway, the primary and most abundant metabolite of the actual plasticizer DEHP, showed a non-significant trend towards an association with both ASD and ADHD. None of the other glucuronidation pathways showed any signs of a relationship to either ASD or ADHD. BPA and DEHP are chemically similar so some overlap in the efficiency of glucuronidation pathways, even if not always statistically significant is not surprising. Approach #1 shows that there is a unique association between compromised detoxification of the plasticizers, BPA and MEHP to neurodevelopmental disorders. Approach #2 confirms this finding.

The figures present a second, visual approach for understanding a wealth of metabolomic data addressing the same. This time instead of only comparing the 12 glucuronidation pathways against each other for evidence of a relationship to disorders, we examined the relationship of the 12 glucuronidation pathways to the metabolome and whether those relationships were associated with clinical diagnosis. Again, interaction between ASD and ADHD parallel each other and show a pattern not shown by any of the other pathways. Specifically, 90+% of the significant p_s_s in the ASD and ADHD groups were positive for the BPA and MEHP pathways.

**Fig 3:**
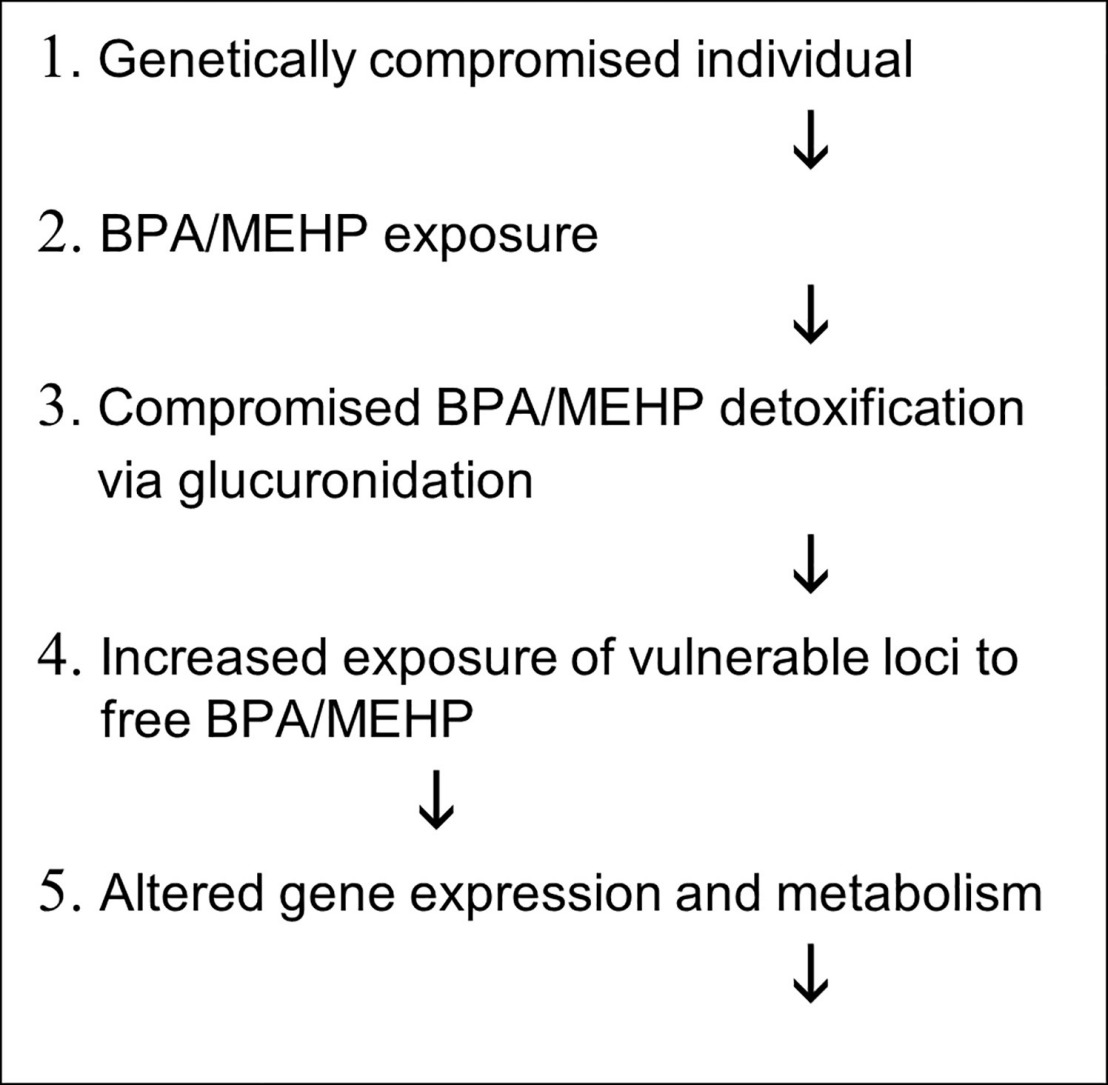
Suggested pathway between BPA and MEHP exposure and ASD/ADHD.

The metabolome reflects metabolism within the body. Its relationship to plasticizer exposure should be the same for all three groups of children. For BPA the difference from the control children is particularly striking; the correlations for control children go in the opposite direction than the children with neurodevelopmental disorder. For MEHP the figure differs in one important way, this time nearly all the control correlations go in the same direction as ASD and ADHD. For ADHD and ASD there is an association between disease and compromised plasticizer detoxification. Based on what is known about the etiology of the two neuro-developmental disorders, the simplest and most direct pathway for BPA and DEHP to lead to neurodevelopmental disorder is the scheme outlined in Fig 3.

It is generally accepted that the etiology of ASD is multifactorial and the most likely mechanism involves environmental toxicants acting upon genetically susceptible individuals [1–7]. We are proposing that the link between steps 2 and 4, step 3 is the compromised glucuronidation of plasticizers in children with ASD and ADHD.

This does occur in humans. A particularly relevant example is a study by Luo et on the relationship between UGT polymorphisms and environmental endocrine disruptors (including BPA) levels in Polycystic Ovary Syndrome (PCOS, [49]). The glucuronidation of BPA is known to be mediated mainly by UGT2B15, with other UGTs (UGT1A1, UGT1A3, UGT1A9, UGT2B4 andUGT2B7) contributing to a lesser extent [49, 50]. Luo at al. found PCOS to be associated with a different distribution of UGT’s and one SNP in particular to be associated with an increased risk of PCOS. Many genes have been found to associated with ASD. Amongst them are the above mentioned UGT’s [51].

The metabolic consequence of this greater residence time in the body of the active forms of the two plasticizers is more exposure to the tissues of the two plasticizers. Elevated blood levels of BPA and MEHP for children with ASD and ADHD have been reported [6, 52–55]. However while consistent with, it does not necessarily follow that this is due to decreased glucuronidation efficiency although elevated blood concentrations are consistent with the impaired glucuronidation hypothesis because the expected result is higher blood concentrations.

This is not likely to be without consequence. Numerous studies have shown both function as endocrine disruptors [17–20]. Endocrine disruption is a very broad term that can encompass multiple metabolic processes ranging from altered gene expression to altered oxidative stress. In rodent models MEHP and BPA impede fetal growth, development, and behavior [56–60]. For ADHD there is direct evidence of a systemic difference in metabolism from control children. The whole body protein synthesis and breakdown rates (turnover) and amino acid flux are elevated in children with ADHD [61] and the BMR is increased [62]. Protein turnover is a systemic process accounting for about 20% of the BMR and directly related to energy expenditure as well as nucleotide metabolism. A difference in the whole-body protein synthesis rate will reflect underlying differences in a significant proportion of the numerous metabolic processes that lead to the synthesis and degradation of proteins. There is no analogous kinetic whole-body data available for ASD even though there is a very extensive literature on the association of multiple specific differences in intermediary metabolism with neurodevelopment disease which is consistent with a systemic response [5–7, 63].

Almost certainly there are other pathways that lead to ASD and ADHD. The scheme presented in Fig 3 applies only to the plasticizer associated pathway. How important plasticizer originated neurodevelopmental disorder is in the overall occurrence of these disorders is not known, but it must account for a significant proportion or would not have been so easy to detect in a metabolic study of moderate size such as this study.

## Strengths of this study

The strengths of this study are: (i) Provides biochemical data independent of epidemiology confirm prior epidemiological studies of associations between the common plasticizers and two common neurodevelopmental diseases. (ii) Fills a gap in present understanding by ‘identifying’ a plausible link (compromised glucuronidation) between genetics, plasticizer exposure and endocrine disruption. (iii) Supports this identification by two semi-independent methods. Firstly, by showing that compromised glucuronidation of BPA is associated with both ASD and ADHD. MEHP showed a non-significant trend. Secondly, documenting a unique relationship between glucuronidation efficiency and the metabolome. Finally, there is nothing new about the efficiency of glucuronidation affecting metabolism and being associated with disease, what is new is that we show this is also a plausible mechanism for ASD and ADHD.

## Limitations of this study

- Children with ASD and ADHD are clinically and biologically diverse. The diagnosis for this study was made on the basis of behavioral criteria. Medication intake could potentially impact upon the regulation of the glucuronidation pathway(s). Despite this confounder, ASD and ADHD groups had significantly reduced capacity in glucuronidation of BPA, which reinforces the salient results present here. Future studies with stricter inclusion criteria are required to confirm the present findings.
- The principal measured parameter in this study was the % of the total compound that is glucuronidated at the time the urine specimen is collected. We equate this as also being a measure of the efficiency of glucuronidation over time from the urine collection for analysis and the immediate prior specimen.
- The number of subjects is relatively small and measured at only one point in time. However it was adequate to obtain interpretable statistically significant data.
- The glucuronidation pathway is the major route for xenobiotic elimination, whereas sulfation is a minor pathway accounting for <10% [22–25]. We have no data on how neurodevelopmental disease affects the sulfation pathway.
- We have no reliable measurement of exposure to plasticizers. However, there is no reason to suspect any major differences between the three groups of children. The children were all drawn from a common environment, the 24 hr. dietary recall data did not reveal any gross differences and the effect sizes in this study were large. Plots of urine total plasticizer amounts against the efficiency of glucuronidation showed no correlations.
- It is possible that the causative agents are not BPA and MEHP. Rather BPA and MEHP serve as markers for other unknown compound(s) that are metabolized by the same BPA and MEHP pathways.

## Conclusion

The two major neurodevelopmental disorders in children are ASD and ADHD. The two disorders are clinically and metabolically different from control children but share an association with compromised detoxification pathways for the plasticizers, BPA and MEHP.

## Supporting information

S1 Dataset (XLSX)

## Abbreviations

ASD: Autism Spectrum Disorders
CTR: Control
ADHD: Attention Deficit/Hyperactivity Disorder
BPA: Bisphenol-A
DEHP: Diethylhexyl Phthalate
MEHP: Methyl ethylhexyl Phthalate
5-oxo MEHP: mono-(2-ethyl-5-oxohexyl) phthalate
5-OH MEHP: mono-(2-ethyl-5-hydroxyhexyl) phthalate
5-CX MEPP: mono-(2-ethyl-5-carboxypentyl phthalate
GCA: Glycocholic acid
GCDA: Glycodeoxycholic acid
α-CEHC: 2,5,7,8-tetramethyl-2-(2’-carboxyethyl)-6-hydroxychroman
γ- CEHC: 7,8-Trimethyl-2-(beta-carboxyethyl)-6-hydroxychroman
UDPGA: Uridine-5′-diphospho-α-D-glucuronic acid
UGTs: Uridine 5′-diphospho-glucuronosyltransferases
